# Supplementary material for: Triptolide Reduces Neoplastic Progression in Hepatocellular Carcinoma by Downregulating the Lipid Lipase Signaling Pathway
Source: Cancers (Basel). 2024 Jan 27;16(3):550. doi: 10.3390/cancers16030550 (PMC10854634; doi:10.3390/cancers16030550)
Supplement: Supplementary file 1 [file cancers-16-00550-s001.zip › cancers-2779629-supplementary.pdf]

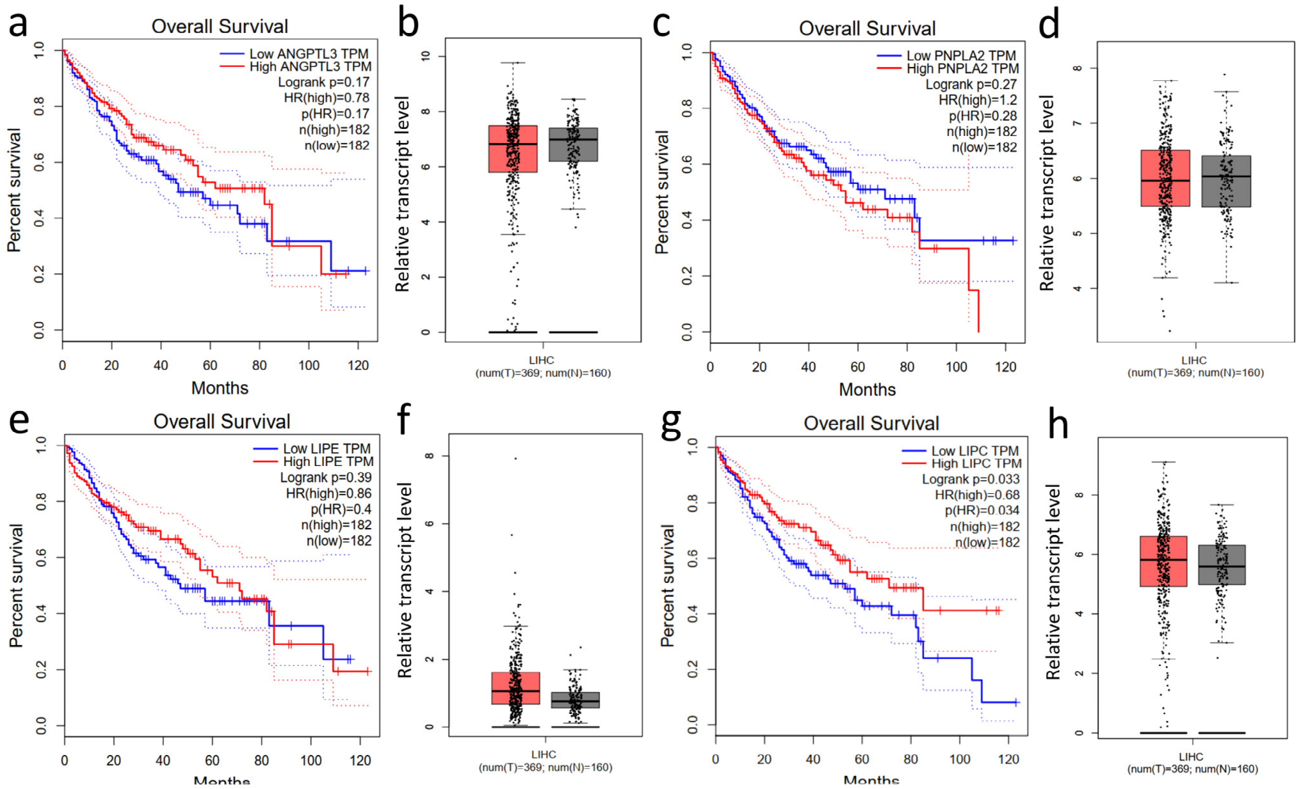

Figure S1 (a) Overall survivals of HCC patients with high ANGPTL3 and low ANGPTL3 were analyzed using Logrank analysis. (b) ANGPTL3 mRNA expression levels in HCC and para-carcinoma tissue in TCGA database. (c) Overall survivals of HCC patients with high PNPLA2 and low PNPLA2 were analyzed using Logrank analysis. (d) PNPLA2 mRNA expression levels in HCC and para-carcinoma tissue in TCGA database. (e) Overall survivals of HCC patients with high LIPE and low LIPE were analyzed using Logrank analysis. (f) LIPE mRNA expression levels in HCC and para-carcinoma tissue in TCGA database. (g) Overall survivals of HCC patients with high LIPC and low LIPC were analyzed using Logrank analysis. (h) LIPC mRNA expression levels in HCC and para-carcinoma tissue in TCGA database.

CAS NO.: 38748-32-2, CDCl3

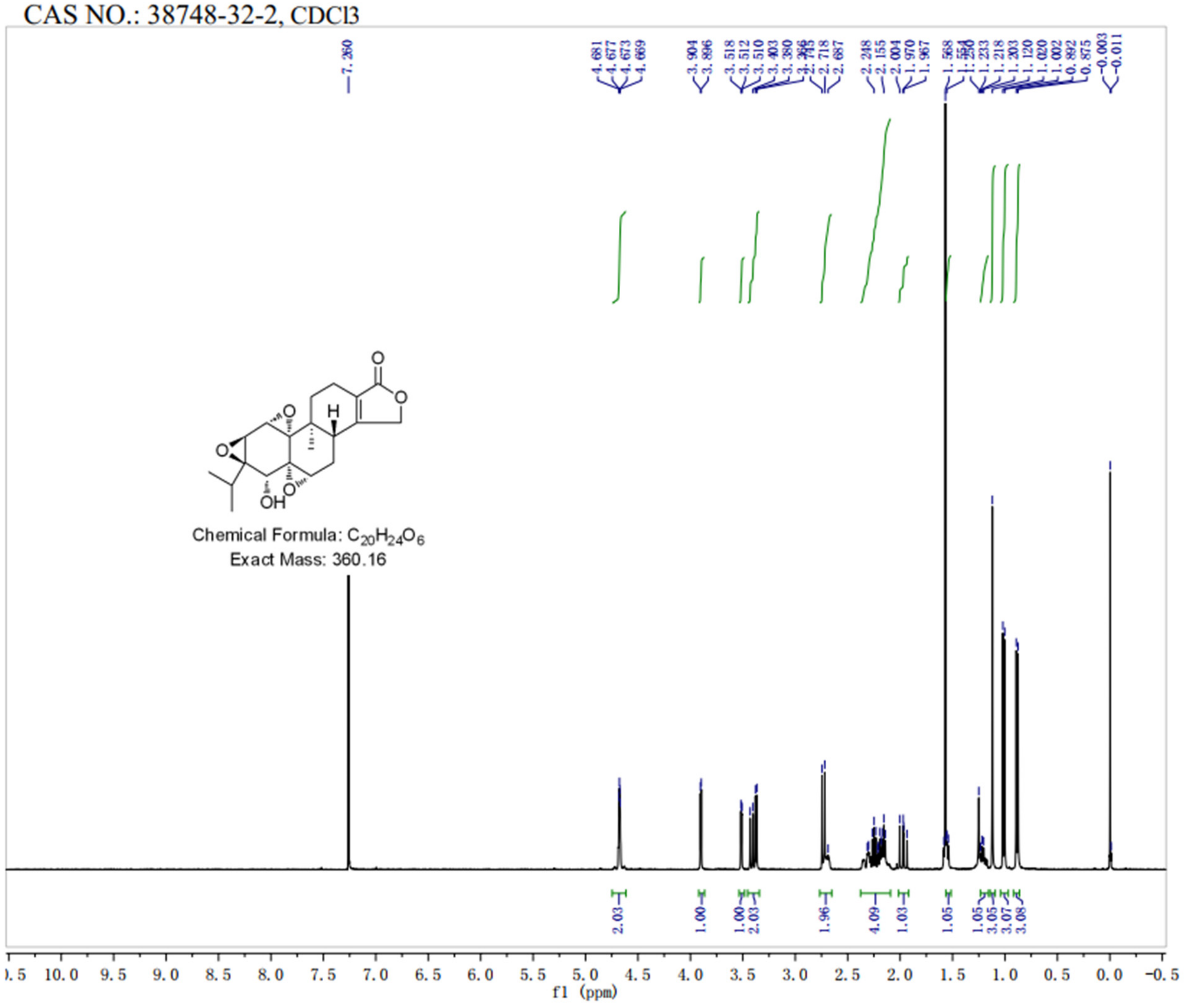

Figure S2 Mass spectrometry of TP.

Table S1 Demographic, clinical and laboratory details of the study cohorts

Characteristic	Patients With HCC (n = 30)	Healthy Control Subjects (n = 30)	*P* value
Male/Female	18/12	15/15	0.604
Age,y	62.4 ± 1.75	62.4 ± 1.90	0.91
BMI (kg/m^2)	22.8 ± 0.59	23.6 ± 0.52	0.34
Smokers	8 (26.7)	7(23.3)	0.766
ALT (IU/L)	51 (11 - 172)	23.5 ± 2.99	0.002
AST (IU/L)	53.1 (18 - 191)	19.1 ± 1.74	< 0.001
Bilirubin (μmol/L)	17.9 (6.57 – 66.49)	13.1 ± 0.65	0.03
GGT (IU/L)	102 (8 - 408)	27.2 ± 4.2	< 0.001
Child-pugh class			
A	25 (83.3)	30	
B-C	5 (16.7)	0	
CRE (μmol/L)	53.8 ± 2.58	61.7 ± 2.35	0.03
BCLC stage			
A1	18 (60)		
A2	3 (10)		
A3	1 (3.3)		
A4	8 (26.7)		
Tumor size			
≥ 3cm	19 (63.3)		
< 3cm	11 (36.7)		
