# Supplementary figures and images for: Triptolide Reduces Neoplastic Progression in Hepatocellular Carcinoma by Downregulating the Lipid Lipase Signaling Pathway
Source: Cancers (Basel). 2024 Jan 27;16(3):550. doi: 10.3390/cancers16030550 (PMC10854634; doi:10.3390/cancers16030550)

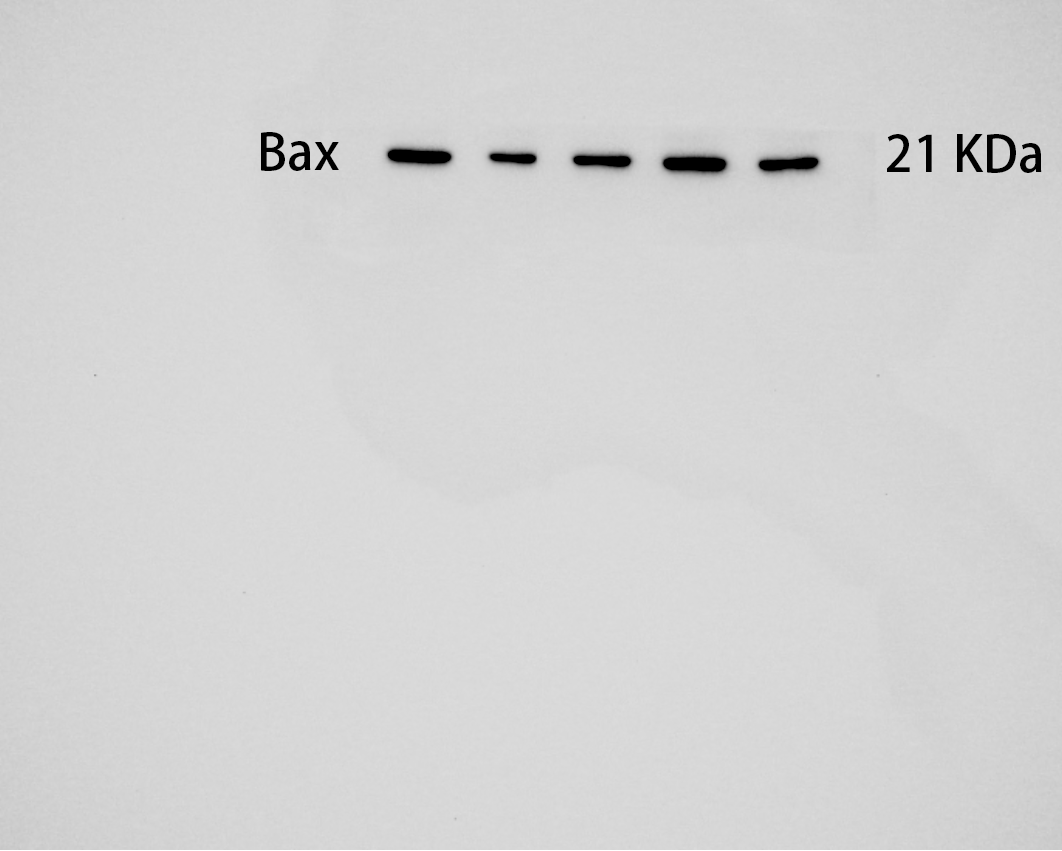

Supplement: Supplementary file 1 [file cancers-16-00550-s001.zip › Western blot datas/Fig.3b BAX.tif]

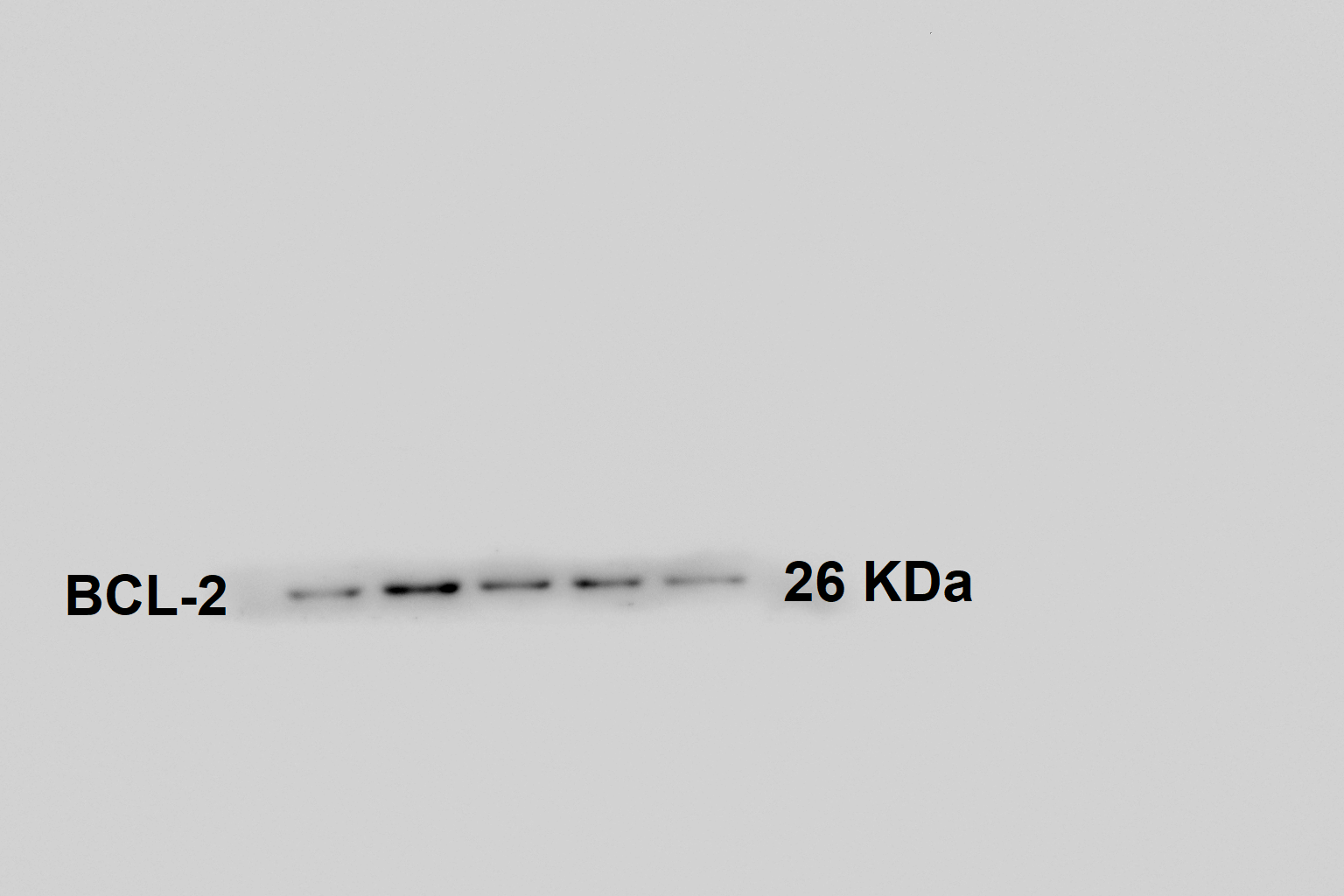

Supplement: Supplementary file 1 [file cancers-16-00550-s001.zip › Western blot datas/Fig.3b BCL-2.tif]

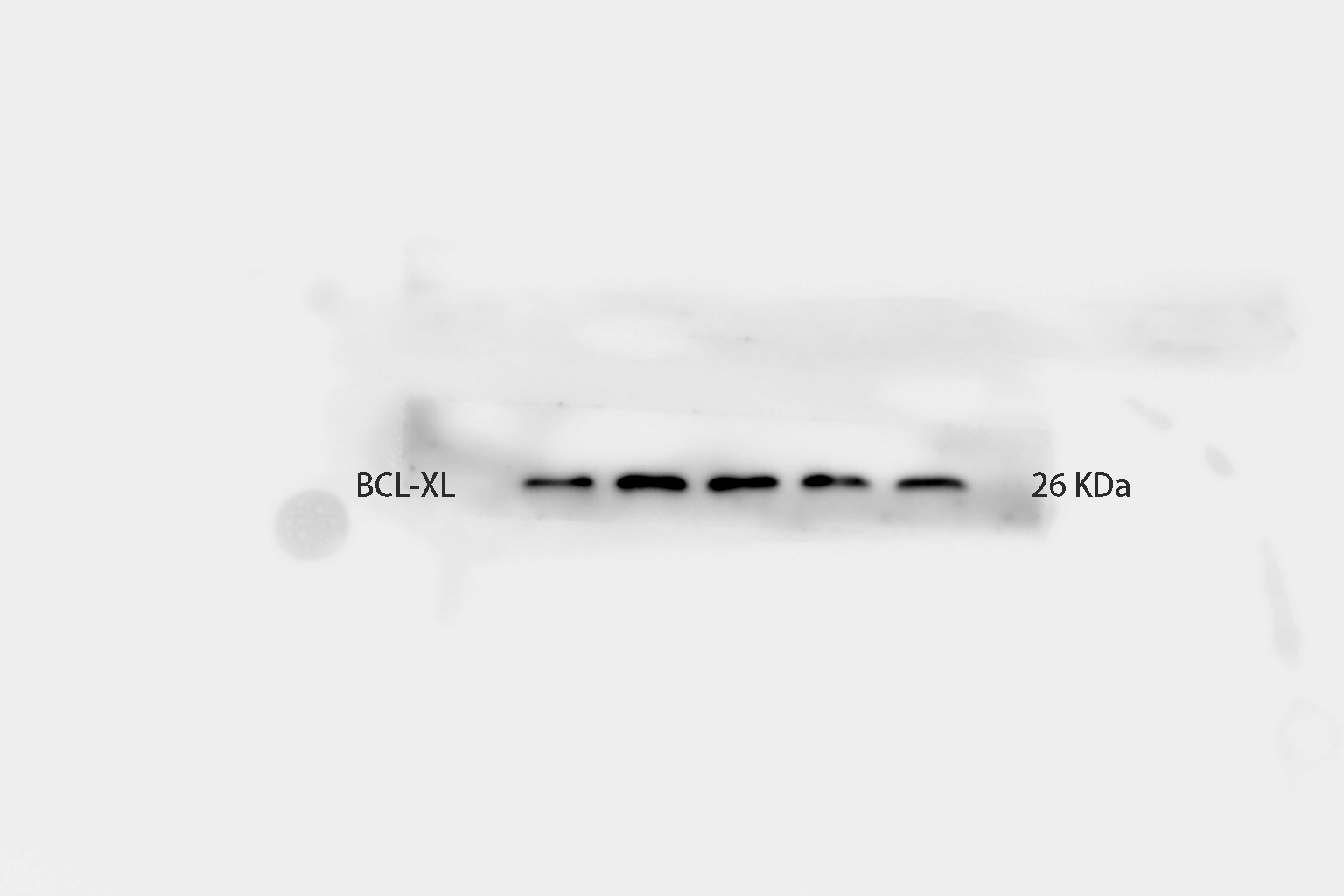

Supplement: Supplementary file 1 [file cancers-16-00550-s001.zip › Western blot datas/Fig.3b BCL-XL.tiff]

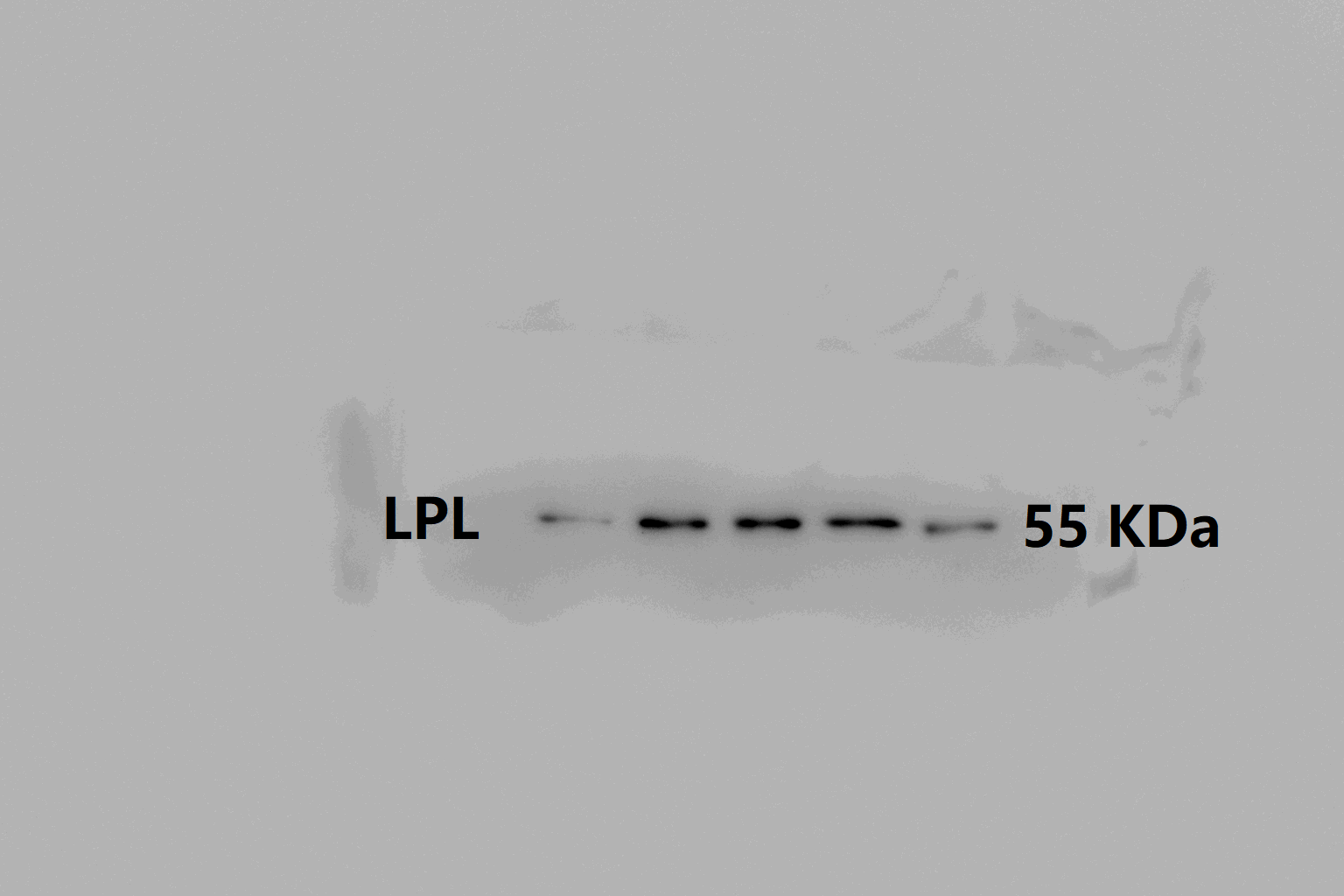

Supplement: Supplementary file 1 [file cancers-16-00550-s001.zip › Western blot datas/Fig.3b LPL.tif]

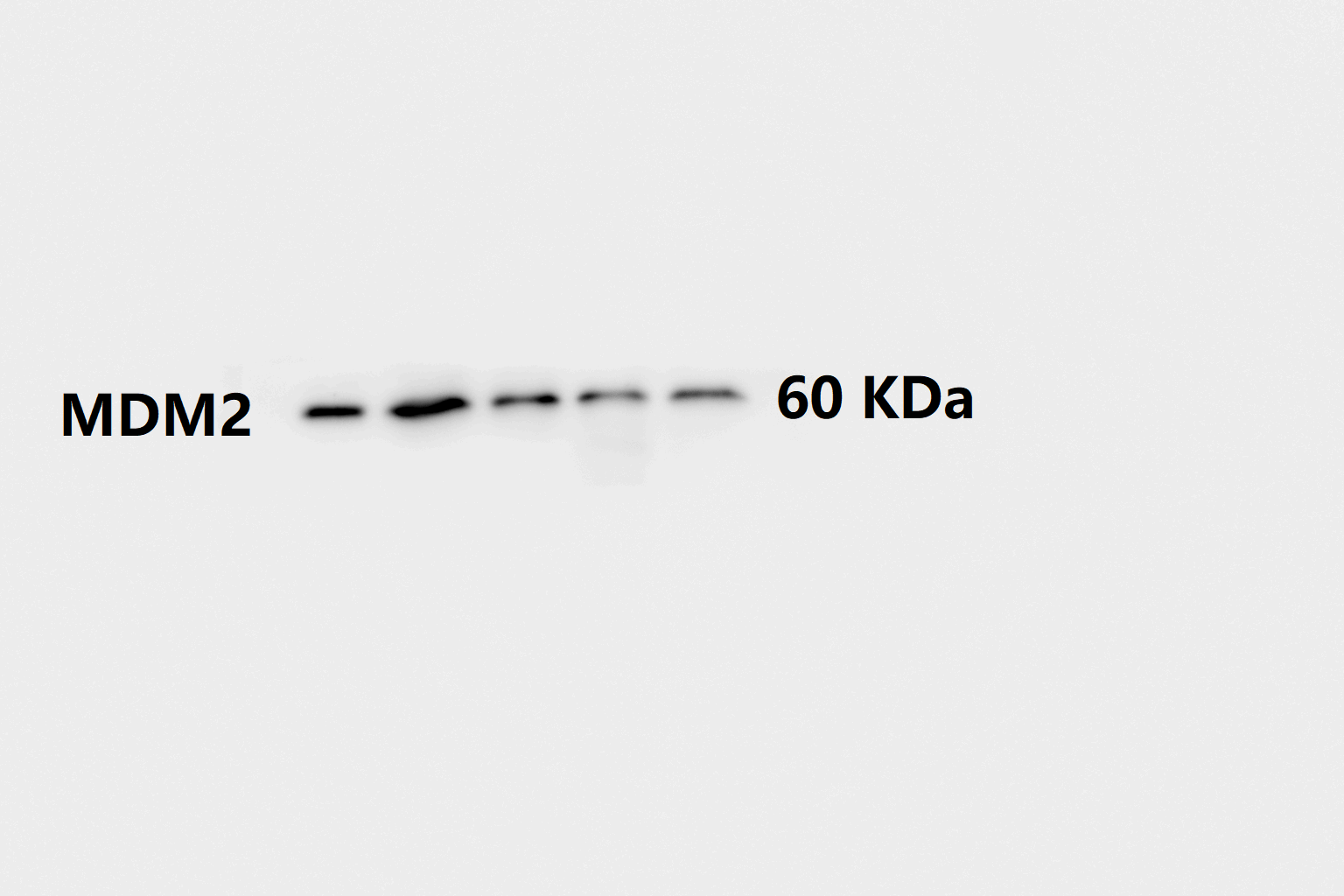

Supplement: Supplementary file 1 [file cancers-16-00550-s001.zip › Western blot datas/Fig.3b MDM.tif]

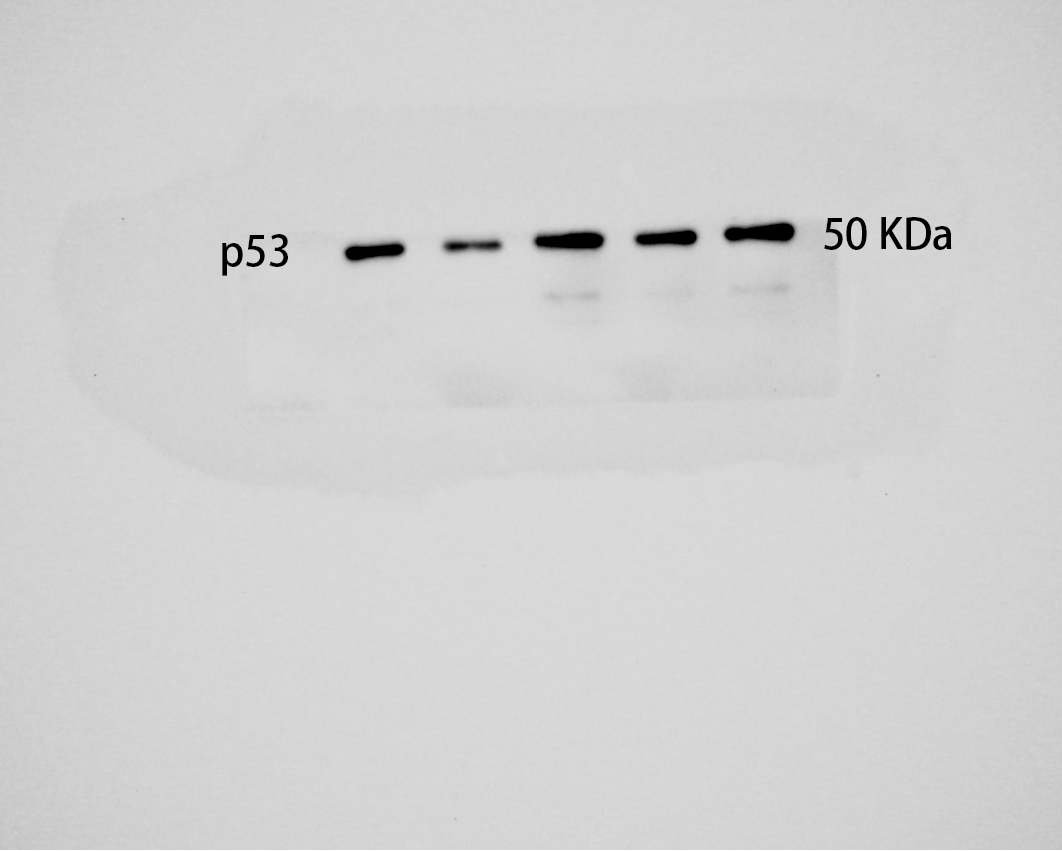

Supplement: Supplementary file 1 [file cancers-16-00550-s001.zip › Western blot datas/Fig.3b p53.tif]

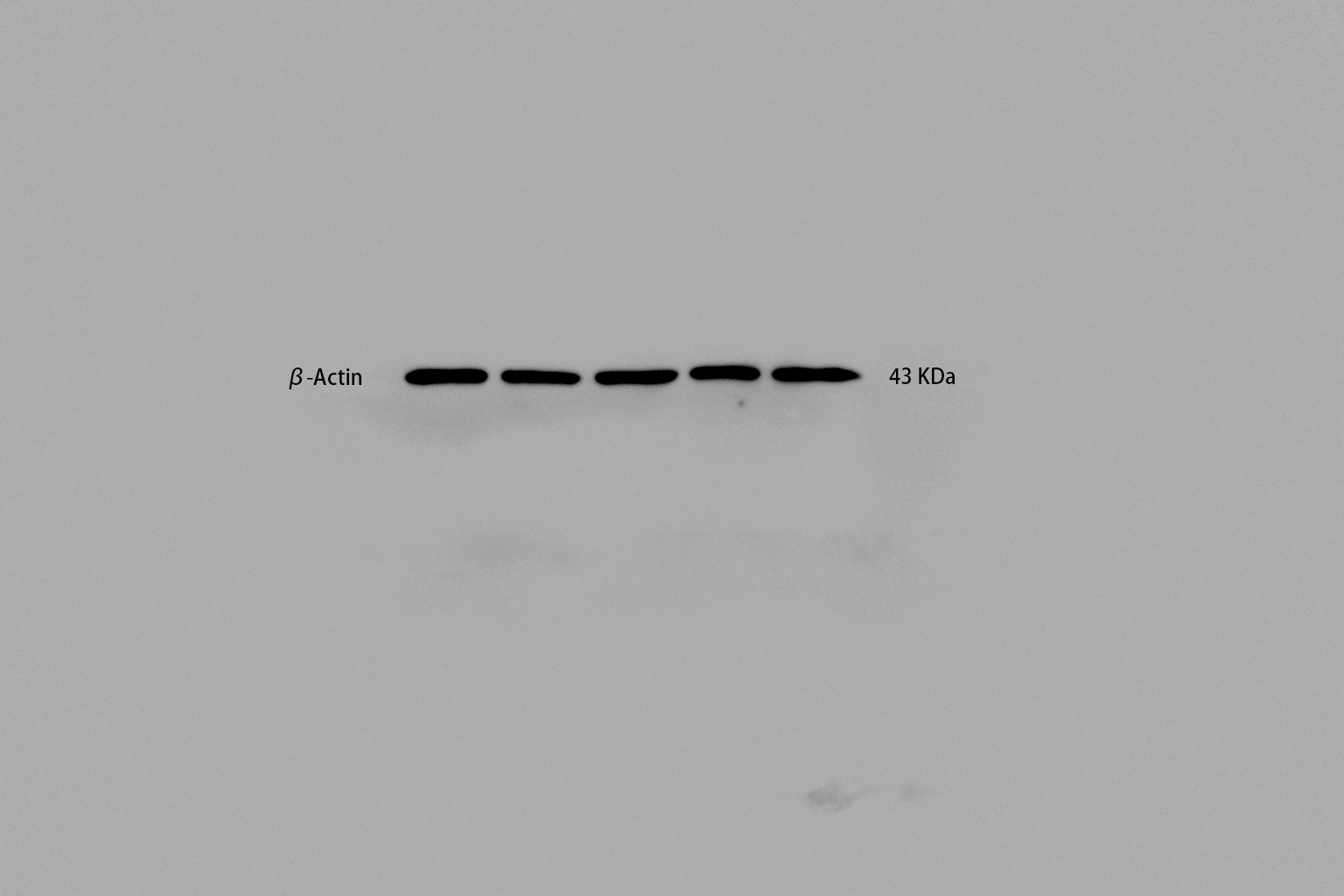

Supplement: Supplementary file 1 [file cancers-16-00550-s001.zip › Western blot datas/Fig.3b a┬-Actin.tiff]

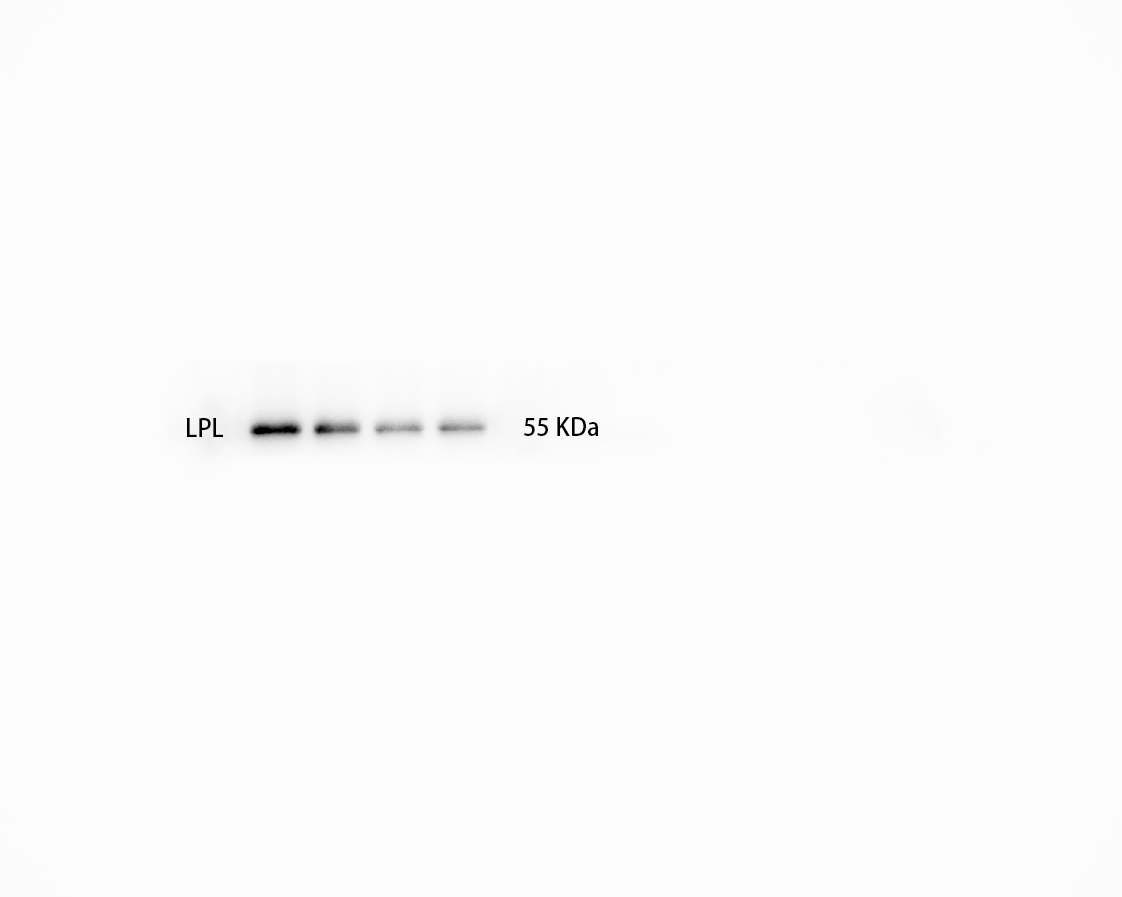

Supplement: Supplementary file 1 [file cancers-16-00550-s001.zip › Western blot datas/Figure.4e LPL.tif]

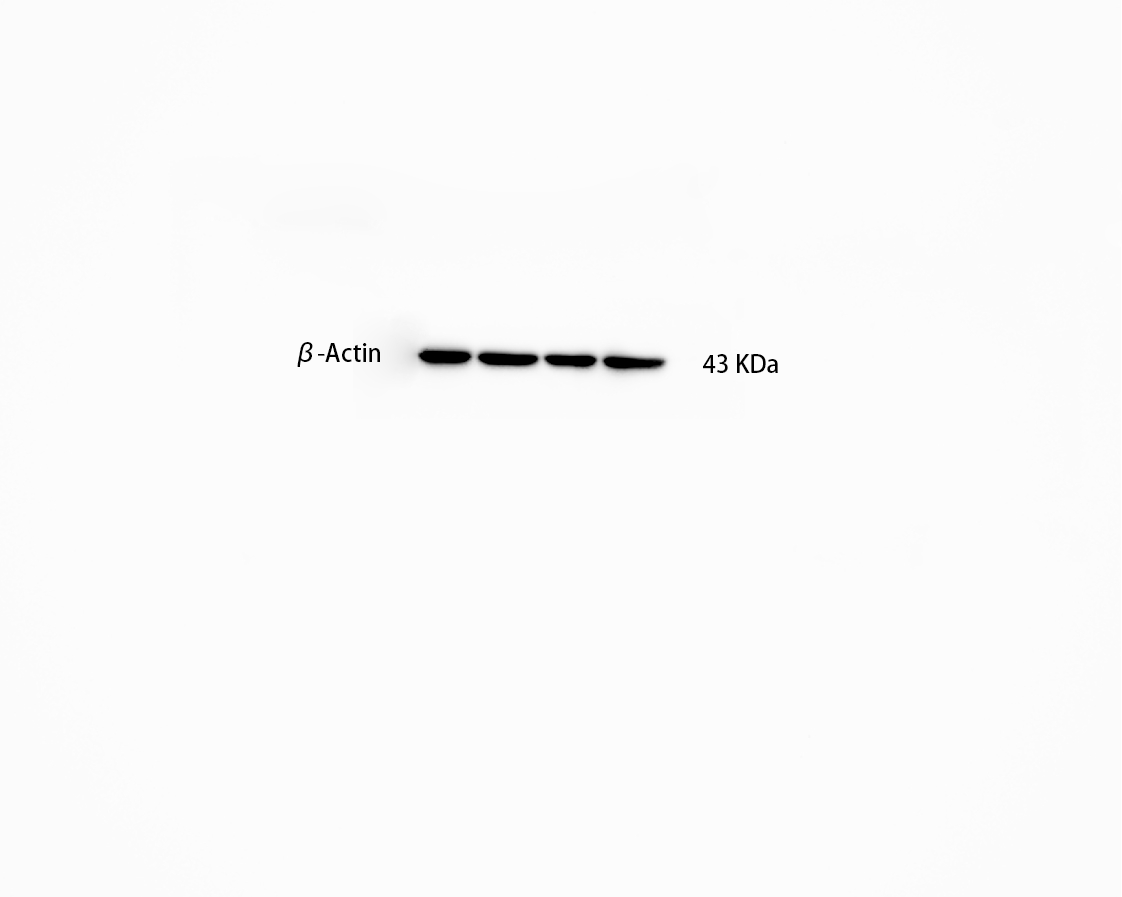

Supplement: Supplementary file 1 [file cancers-16-00550-s001.zip › Western blot datas/Figure.4e a┬-Actin.tif]

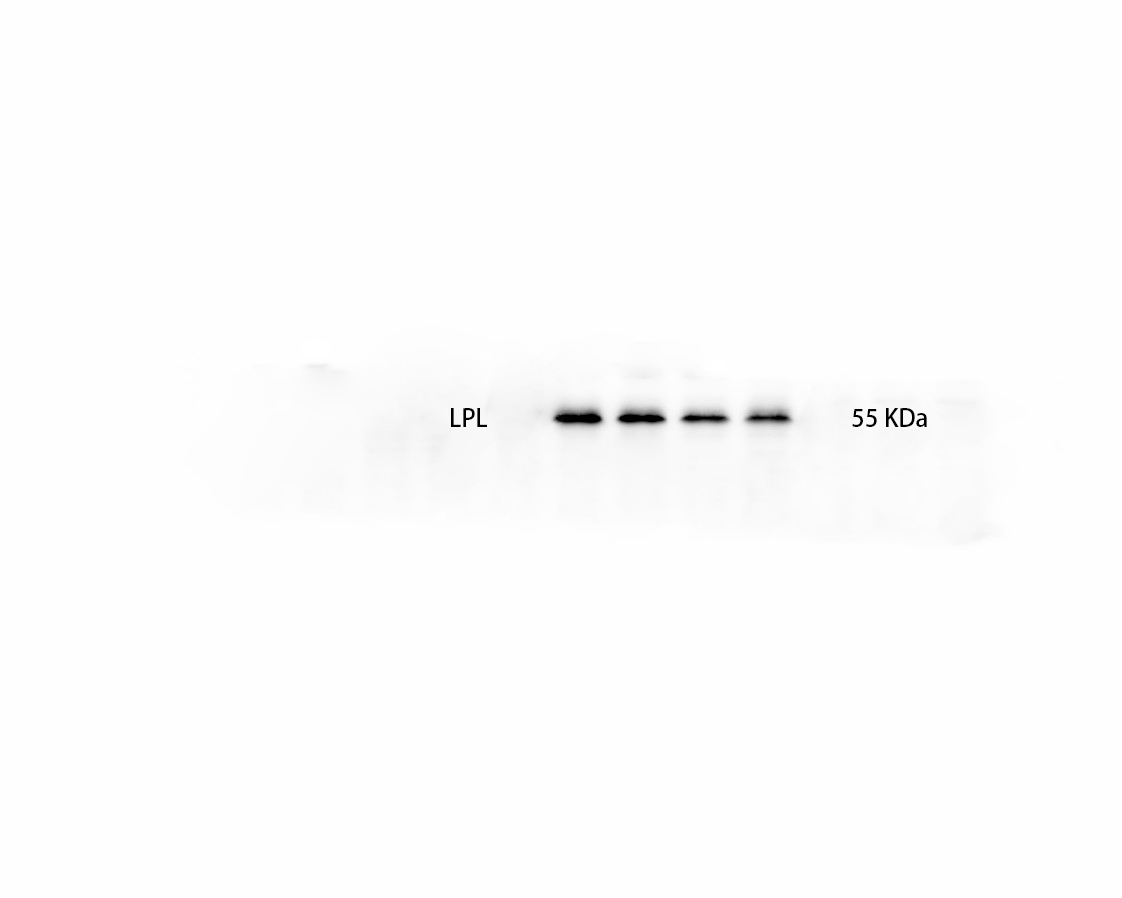

Supplement: Supplementary file 1 [file cancers-16-00550-s001.zip › Western blot datas/Figure.4g LPL.tif]

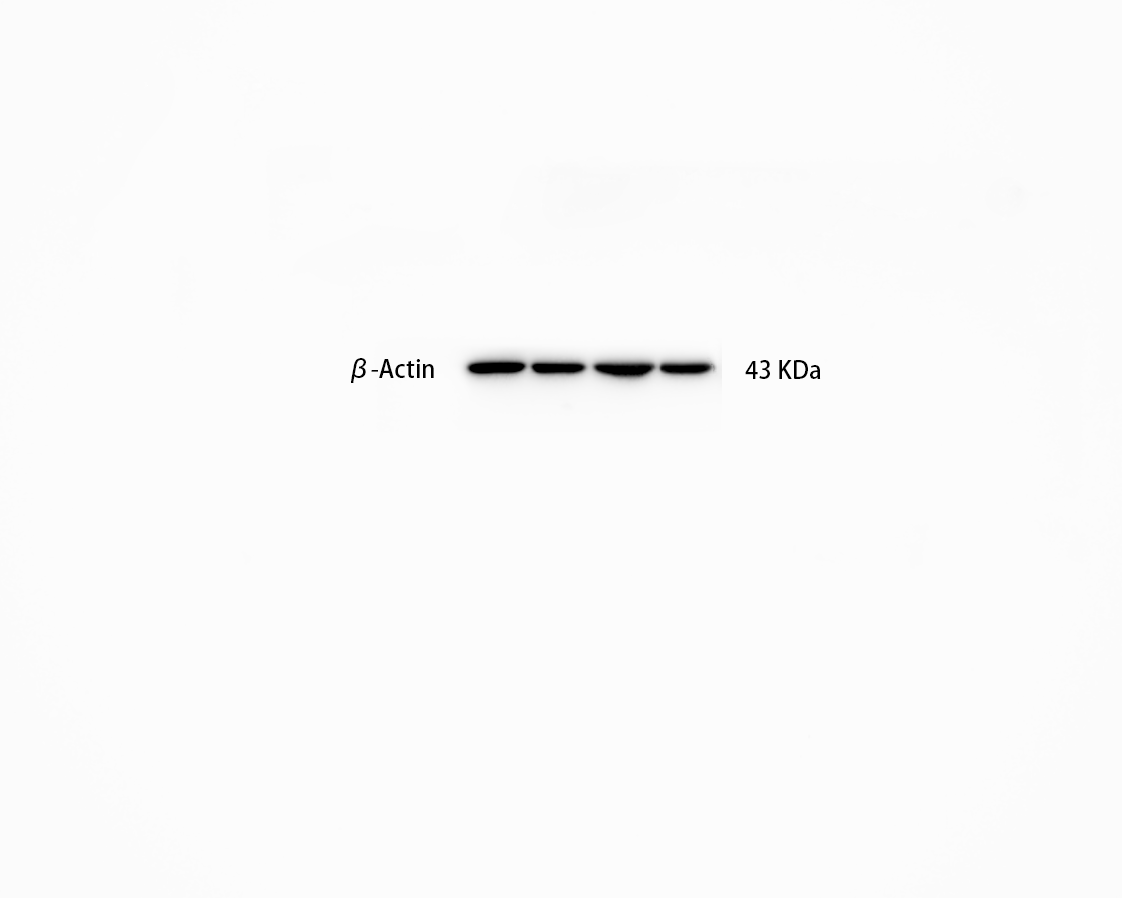

Supplement: Supplementary file 1 [file cancers-16-00550-s001.zip › Western blot datas/Figure.4g a┬-Actin.tif]

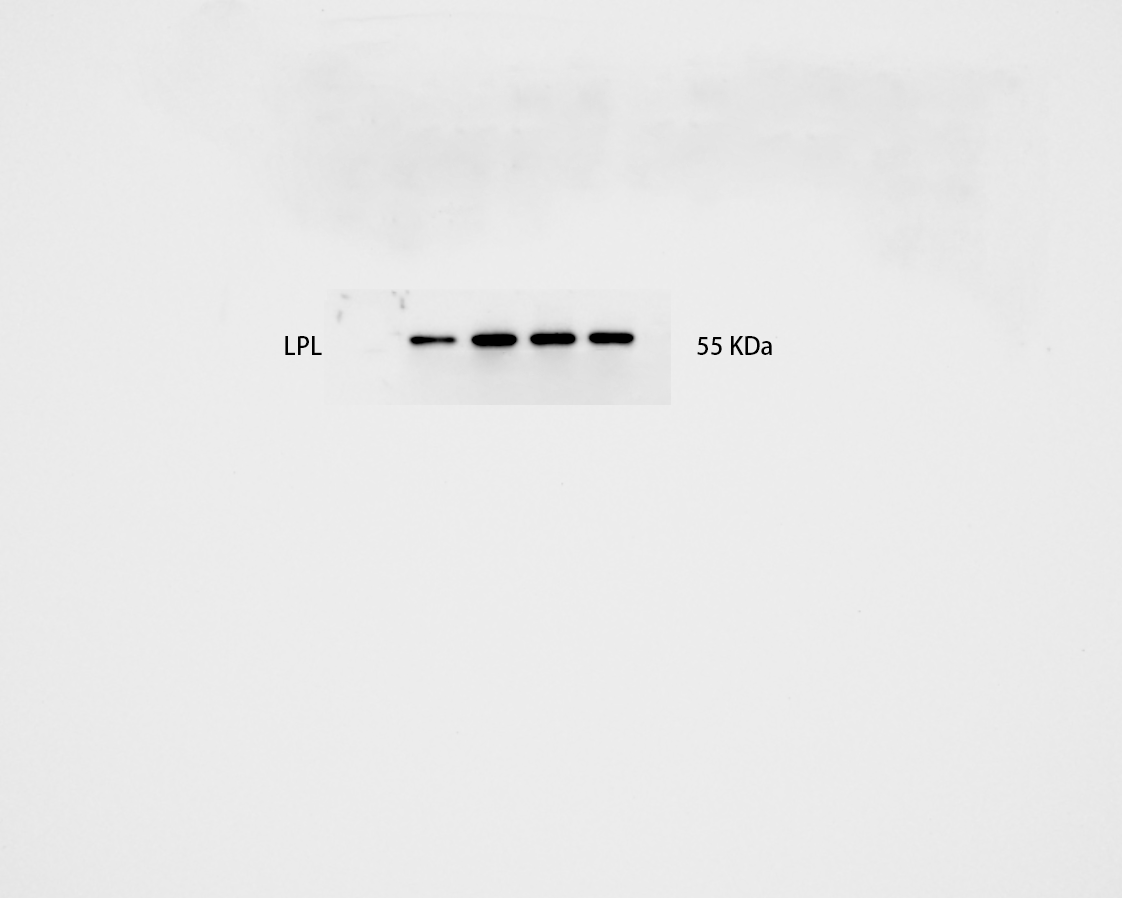

Supplement: Supplementary file 1 [file cancers-16-00550-s001.zip › Western blot datas/Figure.5a LPL.tif]

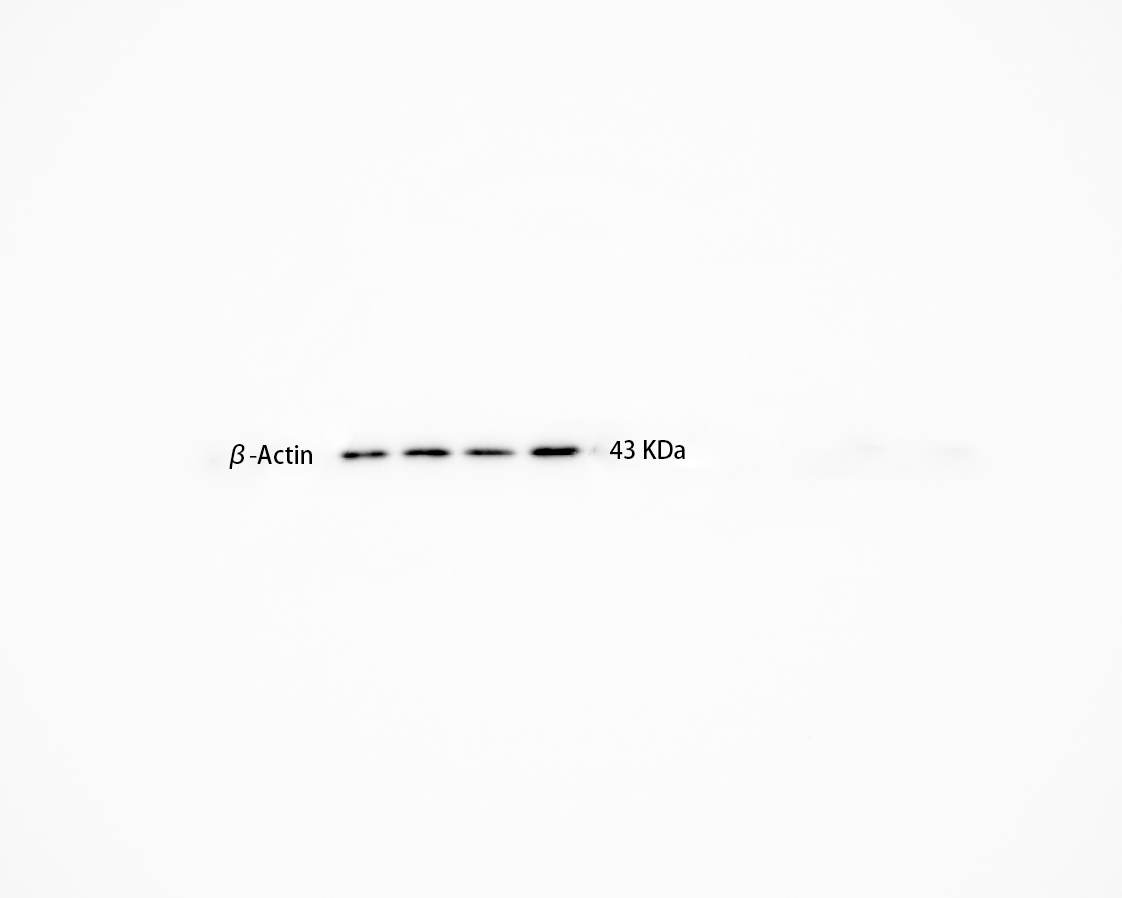

Supplement: Supplementary file 1 [file cancers-16-00550-s001.zip › Western blot datas/Figure.5a a┬-Actin.tif]
